# Different faces of catatonia and how to approach them

**DOI:** 10.1007/s00406-022-01381-0

**Published:** 2022-01-17

**Authors:** Dusan Hirjak, Fabio Sambataro, Georg Northoff, Robert Christian Wolf

**Affiliations:** 1 Department of Psychiatry and Psychotherapy, Central Institute of Mental Health, Medical Faculty Mannheim, University of Heidelberg, Mannheim, Germany; 2 Department of Neuroscience (DNS), Padova Neuroscience Center, University of Padova, Padua, Italy; 3 Mind, Brain Imaging and Neuroethics Research Unit, The Royal’s Institute of Mental Health Research, University of Ottawa, Ottawa, ON Canada; 4 Center for Psychosocial Medicine, Department of General Psychiatry, University of Heidelberg, Heidelberg, Germany

Dear Editor,

We were very pleased to read the excellent article by Parekh et al. [1] who used different functional and structural biomarkers to investigate complex pathomechanisms of acute retarded catatonia. While generally appropriate in its thrust, we would like to reiterate that recently established sensorimotor neuroscience [2, 3] should also consider the following four aspects when examining different clinical manifestations of catatonia:

1. The authors identified both functional hyper- (e.g., sensorimotor, salience, frontoparietal, temporal, and cerebellar regions) and hypoconnectivity (e.g., sensorimotor, temporo-parietal and cerebellar regions) between sensorimotor regions. What seems particularly valuable is that this study also performed multivariate data fusion using multi-set canonical correlation + joint independent component analysis (similar to Hirjak et al. [4]). This analysis revealed a modality-common component for which the mixing coefficients were significantly lower in catatonia patients compared to healthy individuals. The fractional amplitude of low-frequency component spanned multiple brain regions including the bilateral frontal pole, the precuneus, bilateral precentral gyri, lateral occipital cortex, right postcentral gyrus, and the right temporal pole, respectively. The grey matter component encompassed the precuneus, lateral occipital cortex, middle frontal gyrus, superior parietal lobule, precentral gyrus, supramarginal gyrus, right postcentral gyrus, cerebellum, left temporal pole, right frontal pole, bilateral insular cortices, respectively. Looking at these and the previous results from MRI studies on catatonia [2, 5, 6], there is a remarkable correspondence of identified brain regions, particularly key areas of the sensorimotor system. Nevertheless, some seminal pathomechanistic aspects remain poorly understood and are in pressing need of further research, preferably by multimodal and multiparametric studies. In particular, neuroimaging studies focusing on transient changes of functional network connectivity [7] can contribute to a better understanding of both hypo- and hyperkinetic catatonia, because catatonic symptoms can also fluctuate within a few hours or even minutes. To capture such daily or weekly fluctuations, future studies should use multimodal neuroimaging in combination with actigraphy to record sedentary behavior (or psychomotor slowing), monitor activity and behavior, determine energy expenditure, and detect stand and gait as well as capture body position, respectively. Initial studies of this type have already been conducted [8].
2. We appreciate the authors’ transdiagnostic approach that included the examination of patients with a current acute retarded catatonia without being restricted to any ‘other psychiatric diagnosis’. Catatonia is a psychomotor syndrome [5] and now included as an independent diagnostic entity (at the same hierarchical level as schizophrenia or mood disorders) in the 11th revision of the International Classification of Diseases (ICD-11). Clinically, this is highly welcome, since it makes professionals and care-givers more aware of catatonia as one possible psychiatric diagnosis to be considered. This excellent renewal is also an important step to perform more specific neurobiological and clinical (in particular pharmacological) studies on catatonia. A potential limitation, however, is that the authors used only the Bush-Francis Catatonia Rating Scale (BFCRS) and thus were unable to address the affective symptom spectrum in catatonia. In this context, the co-occurrence of motor, affective, and behavioral phenomena needs to be carefully assessed using different rating scales (e.g. Northoff Catatonia Rating Scale [9]), to determine whether and to what extent catatonia is present as it cannot be restricted to just motor-behavioral symptoms. In addition, catatonia is clearly more than just a motor and dopaminergic syndrome and hence, it might be likely caused by aberrant higher-order frontoparietal networks which, biochemically, are insufficiently modulated by gamma-aminobutyric acid (GABA)-ergic transmission (e.g., disbalance between GABA_A(decrease)_ and GABA_B (increase)_ receptor activity) [5].
3. This study of Parekh et al. [1] addressed also the link between neuroimaging biomarkers and treatment response (benzodiazepines and electroconvulsive therapy) in acute retarded catatonia. In this context, the question arises whether it is possible to detect catatonia (similar to other psychiatric disorders) at an early stage before the catatonia becomes an emergency or develops into malignant catatonia? Are there subthreshold or an at risk-syndrome of catatonia? Possible answers to this question could be provided by studies of catatonia in children or adolescents at high clinical risk for psychotic disorders. In these vulnerable populations, the comprehensive assessment of sensorimotor signs, including transient catatonic symptoms, has been unfortunately neglected in the past. In addition, neuroscientists and clinicians alike should also investigate subthreshold catatonic symptoms, because in the absence of flamboyant catatonic symptoms, catatonia might be misdiagnosed, and recommended treatment may be ineffective, or even harmful. Reduced goal-directed and physical activity (including sedentary behavior and reduced gestures [10]), poor appetite, reduced fluid intake, social withdrawal, and selective mutism are transnosological phenomena, but may occur in advance of acute retarded catatonia. Therefore, not only clinicians, but also patients, family members and caregivers should be aware of this issue.
4. The current relevant findings of the deep neural mechanisms of catatonia should be integrated with a complementary thorough description of its clinical presentation and course dating back to the past centuries (similar to Kendler [11] or Foucher et al. [12]). The analysis of historical texts can provide important insights into symptomatological differentiation from other psychiatric disorders and their origins. Especially historical longitudinal studies of unmedicated patients can reveal rich sources of long forgotten clinical expertise and knowledge about spontaneous illness courses. In some circumstances, as it could be impressively seen in the case of catatonia in ICD-11, it can take as long as 150 years of laborious conceptual work to revise nosological classifications back to their roots.
5. Finally, keeping all the above methodological aspects in mind, it is time to create an international, multi-site, large-scale legacy dataset of catatonia patients to examine various demographic, psychopathological, and neurobiological aspects of catatonia and to achieve sufficient statistical power to detect clinically meaningful effects.
